# 2-Amido-3-(1*H*-Indol-3-yl)-*N*-Substitued-Propanamides as a New Class of Falcipain-2 Inhibitors. 1. Design, Synthesis, Biological Evaluation and Binding Model Studies

**DOI:** 10.3390/molecules14010494

**Published:** 2009-01-21

**Authors:** Jin Zhu, Tong Chen, Lili Chen, Weiqiang Lu, Peng Che, Jin Huang, Honglin Li, Jian Li, Hualiang Jiang

**Affiliations:** 1 School of Pharmacy, East China University of Science and Technology, 130 Mei Long Road, Shanghai 200237, P.R. China. E-Mails: jinzh@mail.ecust.edu.cn (J. Z.); libaolu04@126.com (T. C.); luweiqiangnb@126.com (W. L.); magean83@163.com (P. C.); hljiang@ecust.edu.cn (H. J.); 2 Drug Discovery and Design Center, Shanghai Institute of Materia Medica, Chinese Academy of Sciences, 555 Zu Chong Zhi Road, Shanghai 201203, P.R. China; E-Mail: lilichen@mail.shcnc.ac.cn (L. C.)

**Keywords:** 3-(1*H*-Indol-3-yl)-propanamide derivatives, Falcipain-2 inhibitor, Malaria, SAR

## Abstract

The *Plasmodium falciparum* cysteine protease falcipain-2 (FP-2) is an important cysteine protease and an essential hemoglobinase of erythrocytic *P. falciparum* trophozoites. The discovery of new FP-2 inhibitors is now a hot topic in the search for potential malaria treatments. In this study, a series of novel small molecule FP-2 inhibitors have been designed and synthesized based on three regional optimizations of the lead *(R*)-2-phenoxycarboxamido-3-(1*H*-indol-3-yl)-*N*-benzylpropanamide (**1**), which was identified using structure-based virtual screening in conjunction with surface plasmon resonance (SPR)-based binding assays. Four compounds – **1**, **2b**, **2k** and **2l** –showed moderate FP-2 inhibition activity, with IC_50_ values of 10.0-39.4 μM, and the inhibitory activity of compound **2k** was ~3-fold better than that of the prototype compound **1** and may prove useful for the development of micromolar level FP-2 inhibitors. Preliminary SAR data was obtained, while molecular modeling revealed that introduction of H-bond donor or/and acceptor atoms to the phenyl ring moiety in the C region would be likely to produce some additional H-bond interactions, which should consequently enhance molecular bioactivity.

## 1. Introduction

Malaria, which accounts for 300-500 million clinical cases and up to 2.7 million deaths each year. remains one of the deadliest diseases on the planet. About 90 % of these casualties occur in tropical Africa, the great majority being children under the age of 5 [1]. Malaria is caused by protozoan parasites of the genus *Plasmodium*. Of the four species of *Plasmodium* responsible for the disease, *Plasmodium falciparum* is the most lethal. Development of effective vaccines has been significantly hampered by the high mutability of the genome of *Plasmodium falciparum* [2]. The resistance of malaria parasites to available conventional drug therapy is an increasingly serious problem [3,4,5]. Accordingly, the discovery of effective drugs to counter the spread of malaria parasites that are resistant to existing agents, especially those acting on new targets, is an urgent need.

Among various potential new targets, the cysteine protease falcipain-2 (FP-2) of *P. falciparum* is an attractive and most promising target enzyme [6,7]. FP-2 is a principal cysteine protease and essential hemoglobinase of erythrocytic *P. falciparum* trophozoites. Many *in vitro* studies have all confirmed that inhibitors of FP-2 can blocked parasite hemoglobin hydrolysis and halt the development of culture parasites [8,9,10,11,12,13,14,15,16,17,18,19,20,21,22]. Some of them were also effective *in vivo* against murine malaria [15,21,22]. However, FP-2 inhibitors described in the literature are mainly derived from peptide analogues [8,9,10,12,20], which tend to form covalent bonds with the thiolate of the catalytic cysteine and commonly have nanomolar IC_50_ values. Obviously, it is desirable to design non-peptidic inhibitors that would bind non-covalently to the target enzyme, in order to minimize toxicity while retaining the potential for high *in vivo* activity and selectivity. 

Computer-aided drug design has contributed to the introduction of ~50 compounds into clinical trials and to numerous drug approvals [23]. Using Virtual Screening or rational drug design based on homology models, some non-peptidic inhibitors of FP-2 with IC_50_ values in the micromolar range have been reported [14,16,24,25]. Recently, crystal structures for FP-2 have been reported [26,27], and the reservoir of structural and functional information of FP-2 has offered a solid starting point for the rational structure-based design of novel antimalarial drugs targeting for FP-2. By using docking based virtual screening approach in conjunction with a binding assay based on the surface plasmon resonance (SPR), a novel small molecule inhibitor of FP-2 (compound **1**) featuring a 2-amido-3-(1*H*-indol-3-yl)-*N*-aubstitued-propanamide framework has been discovered [28]. After the identification of compound **1** as a possible prototype to design selective inhibitor of FP-2, three regions of the molecule were selected to perform chemical modifications suitable to provide expedient and significant SAR information and improve inhibitory activity. Noticeably, the processes of chemical modifications were achieved step by step from region A to C on the basis of bioassay results. In total, thirteen new compounds including **1** and twelve analogs **2a**-**l** have been synthesized and tested against FP-2. Finally, four compounds – **1**, **2b**, **2k** and **2l** – were found to show moderate inhibitory effects against FP-2, with the inhibitory activity of compound **2k** being ~3 times greater than that of the lead compound **1**. This encouraging result proves the validity of our chemical modification methods. Our molecular modeling results also provided information about the binding between inhibitors and FP-2 and should be helpful for future inhibitor design.

## 2. Results and Discussion

### 2.1. Identification of Binders (Hits) by Virtual Screening

**Figure 1:**
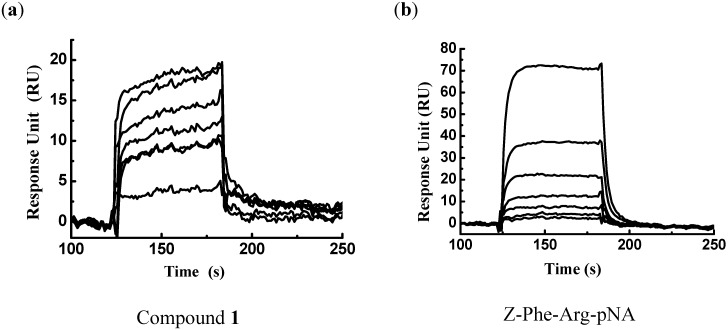
Sensorgrams for binding of compounds **1** (a) and Z-Phe-Arg-pNA (b) to a FP-2 surface on the CM5 sensor chip. Binding responses are shown for **1** and Z-Phe-Arg-pNA injected at concentrations of 0, 1.7, 2.4, 3.3, 4.9, 7 and 10 μM (from bottom to top).

Targeting the crystal structure of FP-2 (PDB entry 2GHU) [26], a total of 80,000 compounds were subsequently docked and ranked according to the Glide and GAsDock software [28]. Finally, 81 compounds were purchased and submitted to biological evaluations against FP-2. Because enzymatic assays are time-consuming, surface plasmon resonance (SPR) measurements were used for the primary screening to determine the binding affinity of these 81 candidate molecules to FP-2. Immobilization of FP-2 on the Biacore biosensor chip resulted in a resonance signal of 9,300 resonance units (RUs). Among the 81 compounds, the biosensor RU of compound **1** was concentration-dependent. The collected data indicated that compound **1** can bind to FP-2 *in vitro*, with a binding affinity towards FP-2 in the micromolar range (*K*_D_ = 5.88 μM). The substrate Z-Phe-Arg-pNA (Bachem AG), which binding affinity to FP-2 is in the submicromolar range (*K*_D_ = 32.1 μM), was used as a positive control. Thus, compound **1** could be designated as a binder (or hit) of FP-2. The binding affinities of compounds **1** and Z-Phe-Arg-pNA are shown in Figure 1a-b, respectively.

### 2.2. Analog Design and Synthesis

**Figure 2:**
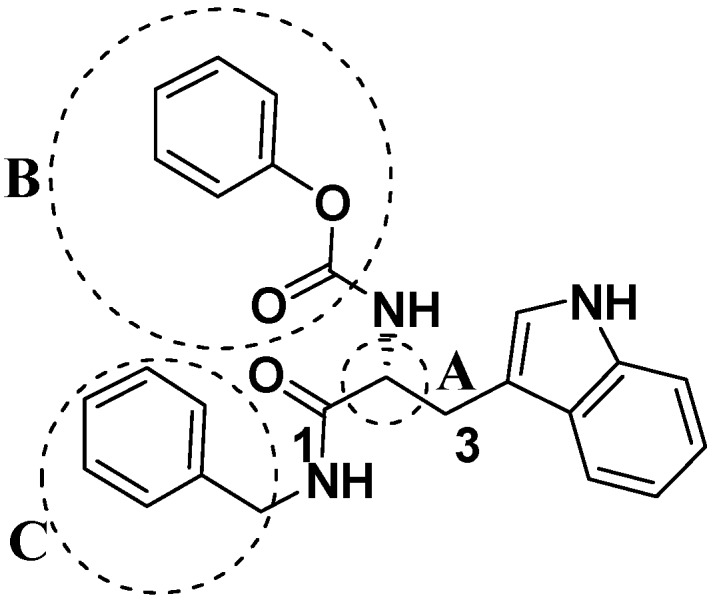
The structure of compound **l** and corresponding three structural modification regions.

Compound **1** (Figure 2) with the higher binding affinities towards FP-2 and identified by surface plasmon resonance (SPR) technology, was used as a lead compound to design new FP-2 inhibitors. Retaining as the common moiety of compound **1** the 2-amido-3-(1*H*-indol-3-yl)-*N*-substituted-propanamide framework, three regions: (A) C_2_ stereo-configuration, (B) the phenoxycarbonyl group, and (C) the N-benzyl substitution (Figure 2), of this molecule were selected to perform chemical modifications that would provide expedient and significant SAR information and improve inhibitory activity. First, we changed the C_2_ stereo-configuration (*R*) in region A to its enantiomer (*S*) and thus obtained analogue **2a.** Secondly, maintaining region A (*R* configuration) and replacing the phenoxycarbonyl group in region B with phenylacetyl and benzyl groups, respectively, we designed compounds **2b-c** (Table 1). Finally, compounds **2d**-**l** were prepared by replacing the benzyl moiety in region C with substituted aryl or aralkyl groups with different electronic and hydrophobic properties..

**Scheme 1a:**
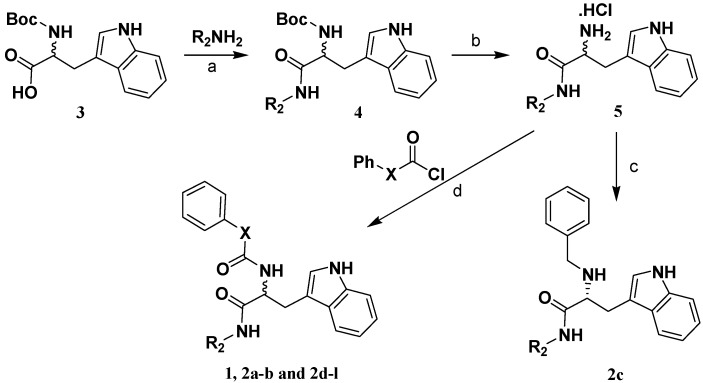
*^a^ Reagents and conditions*: (a) EDCI, Et_3_N, CH_2_Cl_2_, 25 °C; (b) saturated HCl in methanol, 25 °C; (c) (i) benzaldehyde, MgSO_4_, K_2_CO_3_, CH_2_Cl_2_, 25 °C, (ii) NaBH_4_, 0 °C; (d) pyridine, 25 °C; R_2_ is as indicated in Table 1.

Scheme 1 shows the sequence of reactions that led to the preparation of compounds **1** and **2a-l** using *N*-Boc-d-tryptophan or *N*-Boc-l-tryptophan (**3**) as the starting material. 

Compound **4** was prepared by coupling **3** with substituted primary amine using EDCI and Et_3_N in CH_2_Cl_2_. The key intermediate **5** was obtained by deprotection of the *tert*-butyloxycarbonyl group of **4** with a saturated methanolic solution of hydrogen chloride. Then **5** was acylated using different acyl chlorides or benzylated by reductive amination, giving the target compounds **1** and **2a-l**. Synthetic procedures and characterization data are given in the Experimental, while the inhibition rates and IC_50_ values are given in Table 1.

**Table 1:** Chemical Structures of Compounds **1** and **2a**-**m** and Their Inhibitory Activities against FP-2. 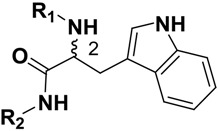

Compd	C_2_ configuration	R_1_	R_2_	Inhibition rate at 10 μM (%)	IC_50_ (μM)
**1**	*R*	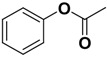	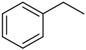	29.4	29.7
**2a**	*S*	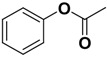	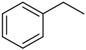	14.3	-
**2b**	*R*	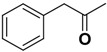	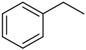	25.7	39.4
**2c**	*R*	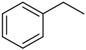	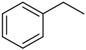	13.0	-
**2d**	*R*	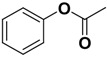	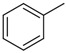	11.8	-
**2e**	*R*	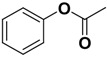	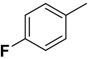	3.1	-
**2f**	*R*	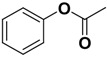	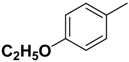	13.6	-
**2g**	*R*	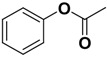	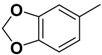	3.1	-
**2h**	*R*	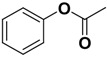	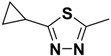	-	-
**2i**	*R*	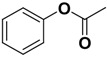	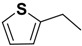	2.2	-
**2j**	*R*	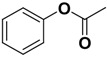	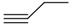	6.6	-
**2k**	*R*	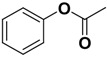	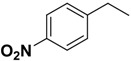	49.5	10.0
**2l**	*R*	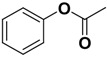	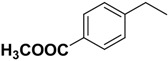	32.9	13.3

### 2.3. Enzyme Inhibition Assay

For the primary assay, the percent inhibitions of the compounds were measured at 10 μM. The results are listed in Table 1. Four compounds, i.e. **1**, **2b**, **2k** and **2l**, showed moderate inhibitory activity (percent inhibition at 10 μM > 20%) and therefore we determined their IC_50_ values, which were 29.7, 39.4, 10.0 and 13.3 μM, respectively. It is clear that the inhibitory activity of compound **2k** was ~3 times higher than that of the lead compound **1**, proving the validity of our chemical modifications. The anti-malarial potential of compound **1** in mice was investigated with *Plasmodium berghei* ANKA using a standard 4-day suppression test [29]. Daily intragastric administration of compound **1** at a dose of 9 mg/kg body weight for the 4 days immediately following infection resulted in an indistinctive (< 50%) reduction in parasitemia relative to normal control (*unpublished data*). The enzymatic activity of compound **1** does not consistently correlate with the animal level efficiency, which may be caused by metabolic instability of the amide groups of compound **1**.

### 2.4. Structure Activity Relationship Correcting with the Binding Models

To gain structural information for further structural optimization, the 3D binding of the designed compounds to FP-2 were superimposed onto the docked conformation of compound **1** by shape similarity. 

**Figure 3:**
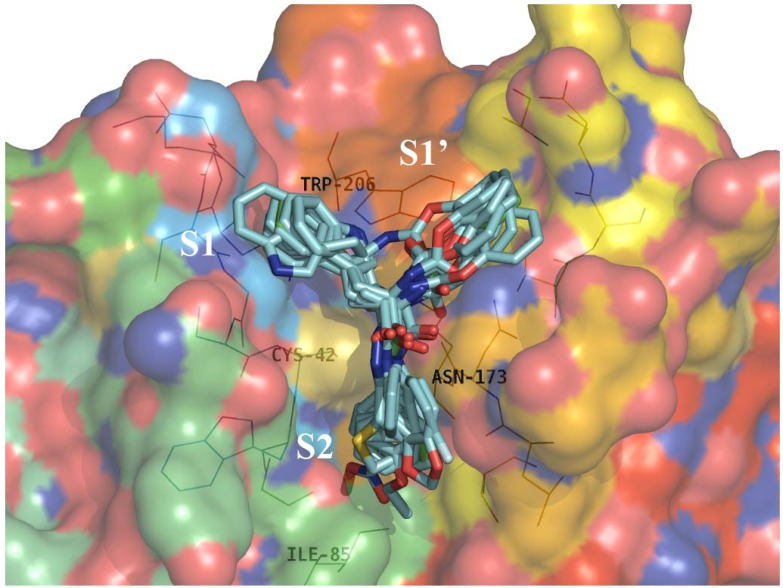
Superimposition of the matched conformations of compounds (**2a**-**2l**, cyan) on compound **1** (green) in the active site of FP-2. Hydrogen atoms have been omitted for clarity. Key residues of the binding pocket are shown as lines. The FP-2 surface was colored by electrostatic potential. The sub-sites are labeled as S1, S1’, and S2. The structure figure was prepared using PyMol (http://pymol.sourceforge.net/).

Figure 3 shows that *R* configuration series of inhibitors reported here (Table 1, **1** and **2b-l**) interact with FP-2 in a similar way: the common indole ring locates on the edge of S1’ and S1 sub-pockets, the phenoxycarbonyl (**1** and **2d-m**), phenacetyl (**2b**) or benzyl moietes (**2c**) fill large hydrophobic region of the S1’ sub-pocket; and the R_2_-substituents (see Table 1) form strong hydrophobic interactions with several hydrophobic residues of the S2 sub-pocket [28]. The inhibitory activities of the less active compounds **2d**-**j** (Table 1) evidently decrease because of the shorter or unsuitable hydrophobic R_2_ groups which separate from the hydrophobic S2 pocket and immerse in the solvent.

**Figure 4:**
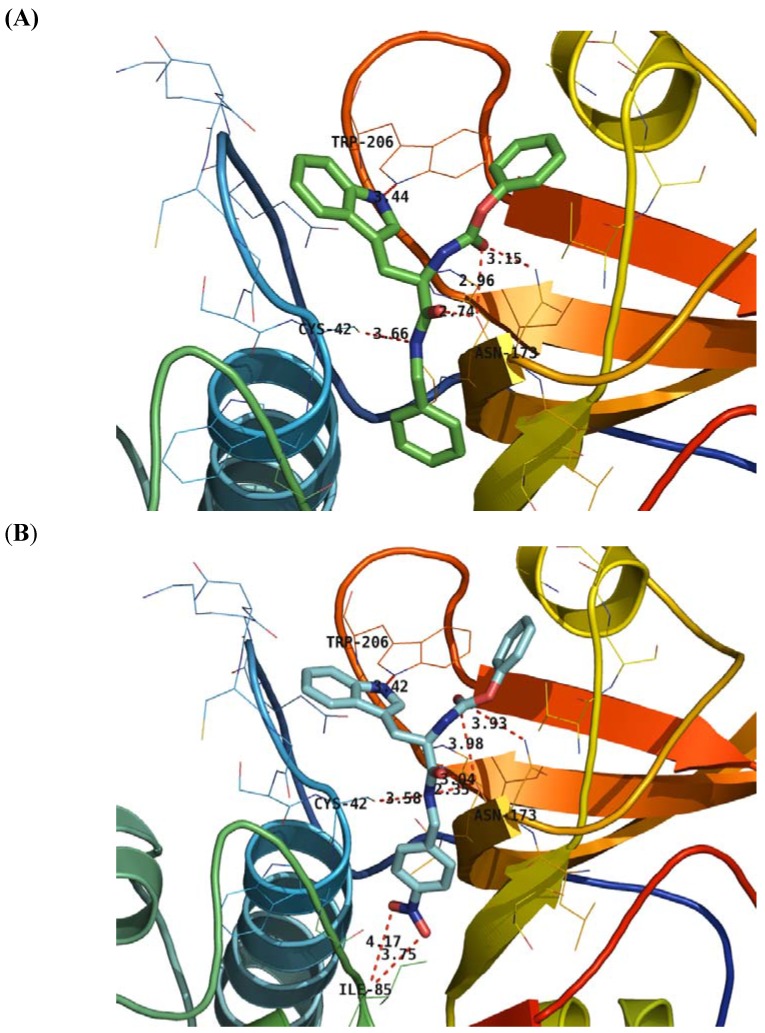
Detailed interactions of **1** (A) and **2k** (B) to the binding sites of FP-2. Compounds 1 and 2k are indicated by cyan and green thick sticks, respectively, and hydrogen atoms have been omitted for clarity. Key residues of the binding pocket are shown as lines. Hydrogen bonds are shown as *red dotted lines* with the distance between donor and acceptor atoms. The structure figures were prepared using PyMol (http://pymol.sourceforge.net/).

More concretely, we compared the 3D binding models of prototype **1** to FP-2 with that of compound **2k** to FP-2 generated based on the docking simulation (Figure 4). Figure 4A shows the nitrogen of the indole ring of compound **1** forms a H-bond with the nitrogen of Trp206, the two nitrogens and three oxygen atoms of the carbamate and amide groups of compound **1** simultaneously form five H-bond interactions with the residues Cys42 and Asn173. From the hydrogen-bond network of compound **1**, we can see that the carbamate moiety is important to maintain the inhibitory activity, explaining why compounds **2b**-**c** lose part of their inhibitory activity (Table 1). The same is true for compound **2a,** whose inverted configuration places it in another orientation. Compared with compound **1**, the nitro group of compound **2k** forms two additional H-bonds with the Ile85 at the end of the subpocket S2 (Figure 4B), which substantially increases the inhibitory activity (Table 1).

## 3. Experimental

Reagents were purchased from Alfa, Acros and the Shanghai Chemical Reagent Company, and used without further purification. Analytical thin-layer chromatography (TLC) was perfomed on HSGF 254 plates (150-200 µm thickness, Yantai Huiyou Company, P.R. China). Yields were not optimized. Melting points were measured in capillary tubes on a SGW X-4 melting point apparatus and are reported without correction. Nuclear magnetic resonance (NMR) spectra were recorded on a Brucker AVANCE 500 NMR instrument. Chemical shifts are given in parts per million (ppm, *δ*) downfield from tetramethylsilane used as internal standard. Proton coupling patterns are described as singlet (s), doublet (d), triplet (t), quartet (q), multiplet (m), and broad (br). Low- and high-resolution mass spectra (LRMS and HRMS) were geberated by electrospray (ESI) produced by a LCQ-TOF spectrometer.

### 3.1. Virtual Screening by Molecular Docking

The crystal structure of FP-2 (PDB entry 2GHU) [26] from *Plasmodium falciparum* was retrieved from the Protein Database Bank [30]. Residues located within 14 Å from the catalytic thiolate of Cys42 were defined as part of the binding site for docking studies. All crystallographic water molecules were removed from the coordinate set. The pipeline of virtual screening based on molecular docking method is presented in detail in [28].

### 3.2. Surface Plasmon Resonance-based FP-2/Ligand Binding Assay

The purification and refolding of recombinant protein FP-2 was performed as described by Shenai *et al* [6]. The binding affinity of compound **1** to FP-2 *in vitro* was determined based on surface plasmon resonance (SPR) technology. The measurement was performed using the dual flow cell Biacore 3000 instrument (Biacore AB, Uppsala, Sweden). Immobilization of FP-2 to the hydrophilic carboxymethylated dextran matrix of the sensor chip CM5 (Biacore) was carried out by the standard primary amine coupling reaction wizard. The FP-2 to be covalently bound to the matrix was diluted in 10 mM sodium acetate buffer (pH 4.2) to a final concentration of 0.069 mg/mL. Equilibration of the baseline was completed by a continuous flow of HBS-EP running buffer (10 mM Hepes, 150 mM NaCl, 3 mM EDTA, and 0.005% (v/v) surfactant P20, pH 7.4) through the chip for 1–2 h. All the Biacore data were collected at 25°C with HBS-EP as running buffer at a constant flow rate of 30 μL/min. All the sensorgrams were processed by using automatic correction for non-specific bulk refractive index effects. The substrate Z-Phe-Arg-pNA (Benzoxycarbonyl-Phe-Arg-p-nitroanilide, Bachem AG) was used for the positive control. The equilibrium constants (*K*_D_ values) evaluating the protein–ligand binding affinities were determined by the steady state affinity fitting analysis of the Biacore data.

### 3.3. Enzyme Inhibition Assay

The purification and refolding of recombinant protein FP-2 was performed as described by Shenai *et al* [6]. IC_50_ values against recombinant FP-2 were determined as described previously [8,31]. FP-2 (30nM) was incubated for 30 min at room temperature in 100 mM sodium acetate, pH 5.5, 10 mM DTT, with different concentrations of tested inhibitors. Inhibitor solutions were prepared from stock in DMSO (maximum concentration of DMSO in the assay was 1%). After 30 min incubation, the substrate Z-Leu-Arg-AMC (benzoxycarbonyl-Leu-Arg-7-amino-4-methylcoumarin, Bachem AG) in the same buffer was added to a final concentration of 25 μM. The increase in fluorescence (excitation at 355 nM and emission at 460 nM) was monitored for 30 min at room temperature with an automated microtiter plate spectrofluorimeter (Molecular Devices, Flex Station). Half-maximal inhibitory concentration (IC_50_) was determined from plots of percent activity over compound concentration using GraphPad Prism software.

### 3.4. Preparation of (R)-2-phenoxycarboxamido-3-(1H-indol-3-yl)-N-benzylpropanamide *(**1**)*.

*Step A: (R)-2-(N-BOC-amino)-3-(1H-indol-3-yl)-N-benzylpropanamide* (**4a**). To a 0 °C stirred solution of the *N*-BOC derivative of (*R*)-tryptophan (0.62 g, 2 mmol) in CH_2_Cl_2_ (10 mL) was added sequentially EDCI (0.58 g, 3 mmol ) and Et_3_N (0.3 g, 3 mmol). After 1 h, the solution was treated with benzylamine (0.22 g, 2 mmol), and then the mixture was allowed to warm to 25 °C. After 2 d of stirring, the mixture was washed sequentially with portions (30 mL) of 5% aqueous HOAc, H_2_O, 5% aqueous NaHCO_3_, H_2_O, and brine, dried, and concentrated. The residue was purified by flash column chromatography on silica gel, eluted with a mixture of EtOAc/petroleum ether (1:2, v/v), to afford **4a** (0.68 g, 84%) as a white solid: mp 138-141 °C; ^1^H-NMR (CDCl_3_) *δ* 1.39 (s, 9H), 3.16 (m, 1H), 3.33 (m, 1H), 4.26 (m, 2H), 4.41 (m, 1H), 6.96 (m, 3H), 7.13 (t, 1H), 7.18-7.21 (m, 4H), 7.35 (d, 1H), 7.66 (d, 1H).

*Step B: (R)-2-Amino-3-(1H-indol-3-yl)-N-benzylpropanamide hydrochloride* (**5b**). Compound **4a** (0.68 g) was dissolved in saturated methanol solution of hydrogen chloride (10 mL). The reaction mixture was stirred at 25 °C for 12 h. Concentration of the reaction mixture to dryness, the residue was washed by Et_2_O, afforded the desired amine hydrochloride (**5b**) (0.50 g, 88%) as a yellow solid: mp 214-218 °C; ^1^H-NMR (CDCl_3_) *δ* 3.16 (m, 1H), 3.25 (m, 1H), 4.02 (s, 1H), 4.27 (m, 2H), 7.00 (t, 1H), 7.07-7.10 (m, 3H), 7.19-7.27 (m, 4H), 7.38 (d, 1H), 7.66 (d, 1H).

*Step C: (R)-2-Phenoxycarboxamido-3-(1H-indol-3-yl)-N-benzylpropanamide* (**1**). A mixture of phenyl chloroformate (82 mg, 0.52 mmol) and compound **5b** (150 mg, 0.46 mmol) in pyridine (5 mL) was stirred 12 h at 25 °C, poured into H_2_O (50 mL), extracted with EtOAc. The combined organic layer was washed, dried, filtered and condensed. The residue was purified by flash column chromatography on silica gel, eluted with a mixture of EtOAc/petroleum ether (1:2, v/v), to afford **1** (95 mg, 50%) as a white solid: mp 176-177 °C; ^1^H-NMR (CDCl_3_) *δ* 3.14 (m, 1H), 3.38 (m, 1H), 4.32 (m, 2H), 4.48 (m, 1H), 5.71 (s, 1H), 5.84 (d, 1H), 6.89 (m, 3H), 7.03 (d, 2H), 7.08 (t, 1H), 7.12-7.18 (m, 5H), 7.29 (m, 3H), 7.68 (d, 1H), 7.91 (s, 1H); ESI-MS *m*/*z* 414 [M+H]^+^; HRMS (ESI) m/z calcd. C_25_H_24_N_3_O_3_ [M+H]^+^ 414.1818, found 414.1813.

### 3.5 Preparation of 2-amide-3-(1H-indol-3-yl)-N-substitued-propanamide compounds ***2a**,**b*** and ***2d**-**l***

These compounds were prepared by the procedure described for the preparation of **1**, with the indicated modifications. 

*(S)-2-Phenoxycarboxamido-3-(1H-indol-3-yl)-N-benzylpropanamide* (**2a**). Replacing *N*-Boc-D-tryptophan with *N*-Boc-l-tryptophan (Step A), compound **2a** was prepared as a white solid: mp 175-176 °C; ^1^H-NMR (CDCl_3_) *δ* 3.21 (q, 1H), 3.46 (q, 1H), 4.26 (q, 1H), 4.34 (q, 1H), 4.55 (q, 1H), 5.74 (s, 1H), 5.90 (d, 1H), 6.95 (s, 1H), 6.98 (m, 2H), 7.10 (d, 2H), 7.15 (t, 1H), 7.19-7.26 (m, 5H), 7.36 (m, 3H), 7.76 (d, 1H), 7.97 (s, 1H); MS (ESI) *m*/*z* 414 [M+H]^+^; HRMS (ESI) m/z calcd. C_25_H_24_N_3_O_3_ [M+H]^+^ 414.1818, found 414.1804.

*(R)-2-(2-Phenylacetamido)-3-(1H-indol-3-yl)-N-benzylpropanamide* (**2b**). Replacing phenyl chloroformate with 2-phenylacetyl chloride (Step C), compound **2b** was prepared as a yellow solid: mp 202-205 °C; ^1^H-NMR (CDCl_3_) *δ* 3.07 (q, 1H), 3.28 (q, 1H), 3.54 (s, 2H), 4.24 (m, 2H), 4.72 (q, 1H), 5.85 (s, 1H), 6.22 (d, 1H), 6.76 (s, 1H), 6.95 (m, 2H), 7.13 (m, 3H), 7.20-7.33 (m, 7H), 7.36 (m, 1H), 7.67 (d, 1H), 7.89 (s, 1H); MS (ESI) *m*/*z* 412 [M+H]^+^; HRMS (ESI) m/z calcd. C_26_H_26_N_3_O_2_ [M+H]^+^ 412.2025, found 412.2022.

*(R)-2-Phenoxycarboxamido-3-(1H-indol-3-yl)-N-phenylpropanamide* (**2d**)**.** Replacing benzylamine with aniline (Step A), compound **2d** was prepared as a white solid: mp 195-197 °C; ^1^H-NMR (CDCl_3_) *δ* 3.28 (m, 1H), 3.53 (q, 1H), 4.71 (m, 1H), 6.00 (d, 1H), 7.07-7.16 (m, 6H), 7.21-7.26 (t, 6H), 7.35-7.42 (m, 3H), 7.80 (d, 1H), 8.12 (s, 1H); MS (ESI) *m*/*z* 400 [M+H]^+^; HRMS (ESI) m/z calcd. C_24_H_22_N_3_O_3_ [M+H]^+^ 400.1661, found 400.1669.

*(R)-2-Phenoxycarboxamido-3-(1H-indol-3-yl)-N-(4-fluorophenyl)-propanamide* (**2e**). Replacing benzylamine with 4-fluorobenzenamine (Step A), compound **2e** was prepared as a yellow solid: mp 185-189 °C; ^1^H-NMR (CDCl_3_) *δ* 3.28 (m, 1H), 3.50 (q, 1H), 4.70 (m, 1H), 6.00 (d, 1H), 6.93 (t, 2H), 7.05-7.17 (m, 6H), 7.21-7.30 (m, 3H), 7.35-7.47 (m, 3H), 7.77 (d, 1H), 8.12 (s, 1H); MS (ESI) *m*/*z* 418 [M+H]^+^; HRMS (ESI) m/z calcd. C_24_H_21_N_3_O_3_ [M+H]^+^ 418.1567, found 418.1572.

*(R)-2-Phenoxycarboxamido-3-(1H-indol-3-yl)-N-(4-ethoxyphenyl)-propanamide* (**2f**). Replacing benzylamine with 4-ethoxybenzenamine (Step A), compound **2f** was prepared as a white solid: mp 180-184 °C; ^1^H-NMR (CDCl_3_) *δ* 1.39 (t, 3H), 3.27 (m, 1H), 3.53 (q, 1H), 3.98 (q, 2H), 4.68 (m, 1H), 6.00 (d, 1H), 6.77 (d, 2H), 7.08-7.17 (m, 7H), 7.20-7.25 (m, 2H), 7.35-7.41 (m, 3H), 7.80 (d, 1H), 8.12 (s, 1H); MS (ESI) *m*/*z* 444 [M+H]^+^; HRMS (ESI) m/z calcd. C_26_H_26_N_3_O_4_ [M+H]^+^ 444.1923, found 444.1938.

*(R)-2-Phenoxycarboxamido-3-(1H-indol-3-yl)-N-(benzo[1,3]dioxol-5-yl)-propanamide* (**2g**). Replacing benzylamine with benzo[1,3]dioxol-5-amine (Step A), compound **2g** was prepared as a white solid: mp 186-188 °C; ^1^H-NMR (CDCl_3_) *δ* 3.26 (q, 1H), 3.51 (q, 1H), 4.67 (q, 1H), 5.93 (s, 2H), 6.00 (d, 1H), 6.44 (d, 1H), 6.64 (d, 1H), 6.92 (s, 1H), 7.05-7.11 (m, 3H), 7.13-7.24 (m, 4H), 7.35-7.41 (m, 3H), 7.60 (d, 1H), 8.13 (s, 1H); MS (ESI) *m*/*z* 466 [M+Na]^+^; HRMS (ESI) m/z calcd. C_25_H_21_N_3_O_5_Na [M+Na]^+^ 466.1379, found 466.1372.

*(R)-2-Phenoxycarboxamido-3-(1H-indol-3-yl)-N-(5-cyclopropyl-1,3,4-thiadiazol-2-yl)-propanamide* (**2h**). Replacing benzylamine with 5-cyclopropyl-1,3,4-thiadiazol-2-amine (Step A), compound **2h** was prepared as a white solid: mp 95-101 °C; ^1^H-NMR (CDCl_3_) *δ* 1.00-1.08 (m, 2H), 1.12-1.14 (m, 2H), 2.21 (m, 1H), 3.38-3.44 (m, 2H), 4.95 (q, 1H), 6.97 (d, 2H), 7.04-7.08 (m, 2H), 7. 15 (q, 2H), 7.20 (d, 1H), 7.30 (m, 3H), 7.61 (d, 1H), 8.02 (s, 1H), 8.12 (s, 1H); MS (ESI) *m*/*z* 448 [M+H]^+^; HRMS (ESI) m/z calcd. C_23_H_22_N_5_O_3_S [M+H]^+^ 448.1443, found 448.1423.

*(R)-2-Phenoxycarboxamido-3-(1H-indol-3-yl)-N-[(thiophen-2-yl)methyl]-propanamide* (**2i**). Replacing benzylamine with (thiophen-2-yl)methanamine (Step A), compound **2i** was prepared as a white solid: mp 184-186 °C; ^1^H-NMR (DMSO-*d*_6_) *δ* 2.98 (q, 1H), 3.16 (q, 1H), 4.29 (q, 1H), 4.46 (m, 2H), 6.91-6.95 (m, 4H), 6.98 (t, 1H), 7.06 (t, 1H), 7. 13-7.22 (m, 2H), 7.29-7.38 (m, 4H), 7.66 (d, 1H); MS (ESI) *m*/*z* 442 [M+Na]^+^; HRMS (ESI) m/z calcd. C_23_H_21_N_3_O_3_NaS [M+Na]^+^ 442.1201, found 442.1200.

*(R)-2-Phenoxycarboxamido-3-(1H-indol-3-yl)-N-(prop-2-yn-1-yl)-propanamide* (**2j**). Replacing benzylamine with prop-2-yn-1-amine (Step A), compound **2j** was prepared as a white solid: mp 173-177 °C; ^1^H-NMR (DMSO-*d*_6_) *δ* 2.93-2.99 (m, 1H), 3.09-3.16 (m, 2H), 3.88-3.91 (m, 2H), 4.27 (q, 1H), 6.92 (d, 2H), 6.98 (t, 1H), 7.06 (t, 1H), 7. 13-7.22 (m, 2H), 7.29-7.35 (m, 3H), 7.66 (d, 1H); MS (ESI) *m*/*z* 384 [M+Na]^+^; HRMS (ESI) m/z calcd. C_21_H_19_N_3_O_3_Na [M+Na]^+^ 384.1324, found 384.1330.

*(R)-2-Phenoxycarboxamido-3-(1H-indol-3-yl)-N-[(4-nitrophenyl)methyl]-propanamide* (**2k**). Replacing benzylamine with (4-nitrophenyl)methanamine (Step A), compound **2k** was prepared as a white solid: mp 182-185 °C; ^1^H-NMR (DMSO-*d*_6_) *δ* 3.26 (q, 1H), 3.46 (dd, 1H), 4.32 (dd, 1H), 4.42 (dd, 1H), 4.62 (m, 1H), 5.82 (d, 1H), 6.06 (s, 1H), 7.01-7.10 (m, 5H), 7.14-7.24 (m, 3H), 7.36 (t, 2H), 7.42 (d, 1H), 7.73 (d, 1H), 8.04 (d, 2H), 8.10 (s, 1H); MS (ESI) *m*/*z* 459 [M+H]^+^; HRMS (ESI) m/z calcd. C_25_H_23_N_4_O_5_ [M+H]^+^ 459.1668, found 459.1677.

*(R)-2-Phenoxycarboxamido-3-(1H-indol-3-yl)-N-(4-methoxycarbonyl-phenylmethyl)-propanamide* (**2l**). Replacing benzylamine with methyl 4-(aminomethyl)benzoate, (Step A) compound **2l** was prepared as a white solid: mp 210-213 °C; ^1^H-NMR (DMSO-*d*_6_) *δ* 3.45 (dd, 1H), 3.48 (dd, 1H), 3.92 (s, 3H), 4.30 (dd, 1H), 4.37 (dd, 1H), 4.59 (m, 1H), 5.86 (m, 2H), 6.98-7.06 (m, 3H), 7.09-7.24 (m, 5H), 7.35-7.45 (m, 3H), 7.75 (d, 1H), 7.90 (d, 2H), 8.04 (s, 1H); MS (ESI) *m*/*z* 494 [M+Na]^+^; HRMS (ESI) m/z calcd. C_27_H_25_N_3_O_5_Na [M+Na]^+^ 494.1692, found 494.1682.

*(R)-2-Benzylamino-3-(1H-indol-3-yl)-N-benzyl-propanamide* (**2c**). A mixture of compound **5b** (250 mg, 0.76 mmol), benzaldehyde (80 mg, 0.76 mmol), K_2_CO_3_ (315 mg, 2.28 mmol), and magnesium sulfate (160 mg) in CH_2_Cl_2_ (5 mL) was stirred at 25 °C for 6 h. Filtration and concentration gave an imine, which was dissolved in 5 mL of methanol. At 0 °C, sodium borohydride (32 mg, 0.84 mmol) was added. After 1 h at 0 °C, the solvent was evaporated and the residue was purified by flash column chromatography on silica gel, eluted with a mixture of EtOAc/petroleum ether (1:2, v/v), to afford **2c** (80 mg, 34%) as yellow solid: mp 38-40 °C; ^1^H-NMR (CDCl_3_) *δ* 3.03 (q, 1H), 3.38 (q, 1H), 3.56-3.60 (m, 2H), 3.70 (d, 1H), 4.44 (d, 2H), 6.95 (d, 1H), 7.00-7.02 (m, 2H), 7.11 (t, 1H), 7.17-7.23 (m, 6H), 7.26-7.33 (m, 4H), 7.37 (d, 1H), 7.60 (t, 1H), 7.66 (d, 1H), 8.04 (s, 1H); MS (ESI) *m*/*z* 384 [M+H]^+^; HRMS (ESI) m/z calcd. C_25_H_26_N_3_O [M+H]^+^ 384.2076, found 384.2072.

## 4. Conclusions

In this study, using a structure-based virtual screening approach in conjunction with chemical synthesis and bioassay we have discovered four moderately active FP-2 inhibitors (**1**, **2b**, **2k** and **2l**). The preliminary SAR data was obtained, which shows that an *R* configuration of C_2_ in A region, a phenoxycarboxamido moiety in the B region and a 4-nitrobenzyl moiety in the C region are essential for the inhibitory activity against FP-2. Meanwhile, analysis of binding positions revealed that introduction of H-bond donor or/and acceptor atom to phenyl ring part in C region would be likely to produce some additional H-bond interactions to enhance molecular bioactivity. Although the most potent compound **2k** at this stage has activity in the submicromolar range, the preliminary structure-activity relationships (SARs) of compounds **2a**-**l** give some valuable clues for further structural optimization. The anti-malarial activities of these FP-2 inhibitors presented here need to be further investigated. 
